# Bio-behavioral synchrony is a potential mechanism for mate selection in humans

**DOI:** 10.1038/s41598-022-08582-6

**Published:** 2022-03-21

**Authors:** Lior Zeevi, Nathalie klein Selle, Eva Ludmilla Kellmann, Gal Boiman, Yuval Hart, Shir Atzil

**Affiliations:** 1 Department of Psychology, The Hebrew University of Jerusalem, Jerusalem, Israel; 2 Department of Education and Psychology, Freie University, Berlin, Germany

**Keywords:** Psychology, Human behaviour, Autonomic nervous system, Social behaviour

## Abstract

The decision with whom to form a romantic bond is of great importance, yet the biological or behavioral mechanisms underlying this selective process in humans are largely unknown. Classic evolutionary theories of mate selection emphasize immediate and static features such as physical appearance and fertility. However, they do not explain how initial attraction temporally unfolds during an interaction, nor account for mutual physiological or behavioral adaptations that take place when two people become attracted. Instead, recent theories on social bonding emphasize the importance of co-regulation during social interactions (i.e., the social coordination of physiology and behavior between partners), and predict that co-regulation plays a role in bonding with others. In a speed-date experiment of forty-six heterosexual dates, we recorded the naturally occurring patterns of electrodermal activity and behavioral motion in men and women, and calculated their co-regulation during the date. We demonstrate that co-regulation of behavior and physiology is associated with the date outcome: when a man and a woman synchronize their electrodermal activity and dynamically tune their behavior to one another, they are more likely to be romantically and sexually attracted to one another. This study supports the hypothesis that co-regulation of sympathetic and behavioral rhythms between a man and a woman serves as a mechanism that promotes attraction.

## Introduction

Humans belong to a limited number of mammalian species who form long-lasting pair bonds with selected sexual partners^1,2^. Unlike parental bonding, pair-bonding is an attachment between two adults that includes a voluntary selection of the partner. Pair bonding in humans is characterized by sexual desire and romantic infatuation^3–5^, followed by a long-lasting attachment and familial cooperation^6,7^. Selecting a romantic partner is a significant process in human life, affecting many aspects of living and well-being^8–10^. Yet, little is known about the bio-behavioral mechanisms underlying this selection in humans^11^.

The assortative process of coupling and sexual choice is referred to as *mate selection*^12,13^. Classic evolutionary theories suggest that females select a male partner based on strength and resources to increase the survival of the offspring, while males are drawn to physical traits that are correlated with fertility^14–16^. More recent theories of mate choice focus on perceived genetic compatibility^17,18^. These explanations focus on static physical features of sexual selection, however, they do not explain the role of social interaction and cooperation in the dynamic process of mate selection^19,20^.

The study of human bonds points to two key aspects of human interactions: dyadic synchronization and dyadic attunement. From a psycho-biological perspective, synchrony is defined as the matching of affective states and biological rhythms in time for the purpose of social regulation^21^. Synchrony was largely studied in mothers and infants as a mechanism for bonding and regulation^22–25^, however, it can serve similar co-regulatory functions in adults as well. In early life, caregivers use synchrony to regulate most aspects of infants’ physiological processes, including metabolism, energy expenditure, immunity, temperature and arousal^25–27^. Physiology is also regulated in adult-adult attachment^28,29^, and this co-regulation is associated with physical health by influencing allostatic balance^30–32^. Moreover, synchrony in electrodermal activity between romantic partners was found to associate with sexual satisfaction^33^, and marital satisfaction^34,35^. Another central feature of dyadic interaction is behavioral attunement to the partner^36–39^. While synchrony refers to the *simultaneous* matching between partners, attunement refers to the *sequential* adjustment of behavior in response to the partner. Behavioral attunement is associated with improved interactive engagement^37^ and social regulation^36^.

Dyadic synchrony and attunement are both *temporal* phenomena of complex social interaction^40,41^, which reflect dyadic co-regulation. Definitions of co-regulation vary in the literature^32^. In this work, we define co-regulation as the levels of the social linkage in oscillations^32^ of sympathetic physiology and motor behavior during interaction between two date-partners. Co-regulation in close bonds is adaptive because it can contribute to regulatory stability for both partners^32^. We hypothesize that both physiological synchrony and behavioral attunement with a potential romantic partner could serve as indicators for successful bonding and thus promote initial sexual and romantic attraction. To test this hypothesis, we measure the extent to which a co-regulation between partners is associated with their attraction to one another.

**Figure 1:**
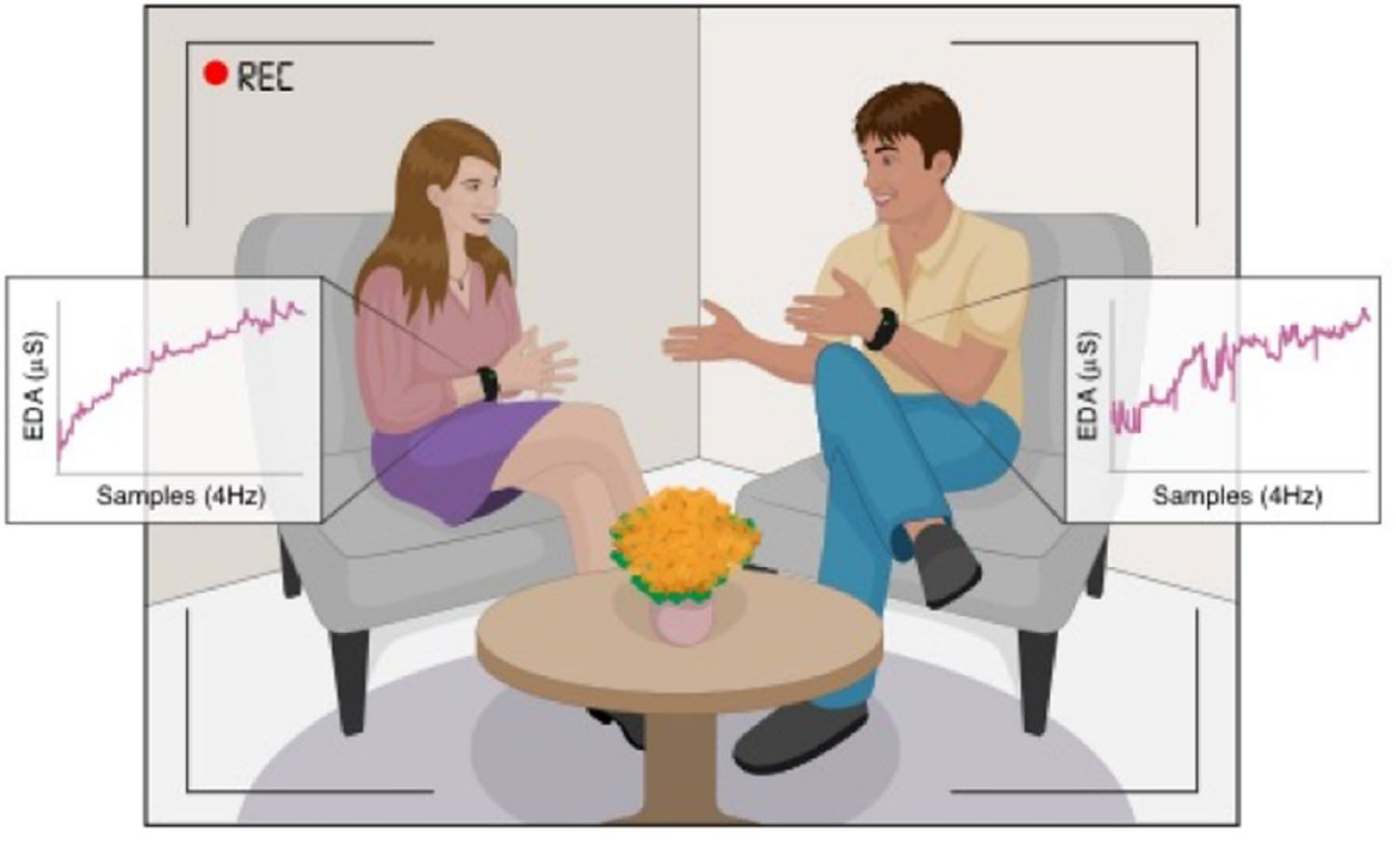
The experimental setting. A man and a woman meet for a speed date while their behavior and physiology are being recorded, providing 1200 physiological data points (sampled at 4 Hz) and 3000 behavioral data points per date for each subject (sampled at 10 Hz). The room design, as well as the ambulatory recording equipment, enabled participants to freely interact, for an ecologically-valid estimation of bio-behavioral measures, naturally occurring during a romantic date. After the date, participants rate their romantic interest and sexual attraction to the partner. We measured the association between physiological synchrony and behavioral attunement during a date, and the romantic and sexual ratings after the date.

In this study, we conducted an ecologically-valid speed-date experiment consisting of forty-six dates which were obtained over three runs, in each run men and women met in a speed-dating rotation. Each date lasted five minutes, during which we recorded the participants’ electrodermal activity, a measurement of the sympathetic nervous system^42,43^, at a frequency of 4 Hz using an Empatica E4 wristband. In parallel, we video-recorded the dates and conducted an automated video-analysis to extract the motion of each partner pixel-by-pixel, frame-by-frame at a frequency of 10 Hz (Fig. 1). We then calculated electrodermal synchrony and motor attunement, by computing cross-correlations between the partners during the date. Immediately after the date, each participant rated their romantic interest in their partner, their sexual attraction to their partner, and the partner’s physical appearance. We tested whether increased electrodermal synchrony and motor attunement between partners during the date is predictive of increased romantic interest and sexual attraction.

## Results

### Electrodermal synchrony during a date is associated with romantic interest

**Figure 2:**
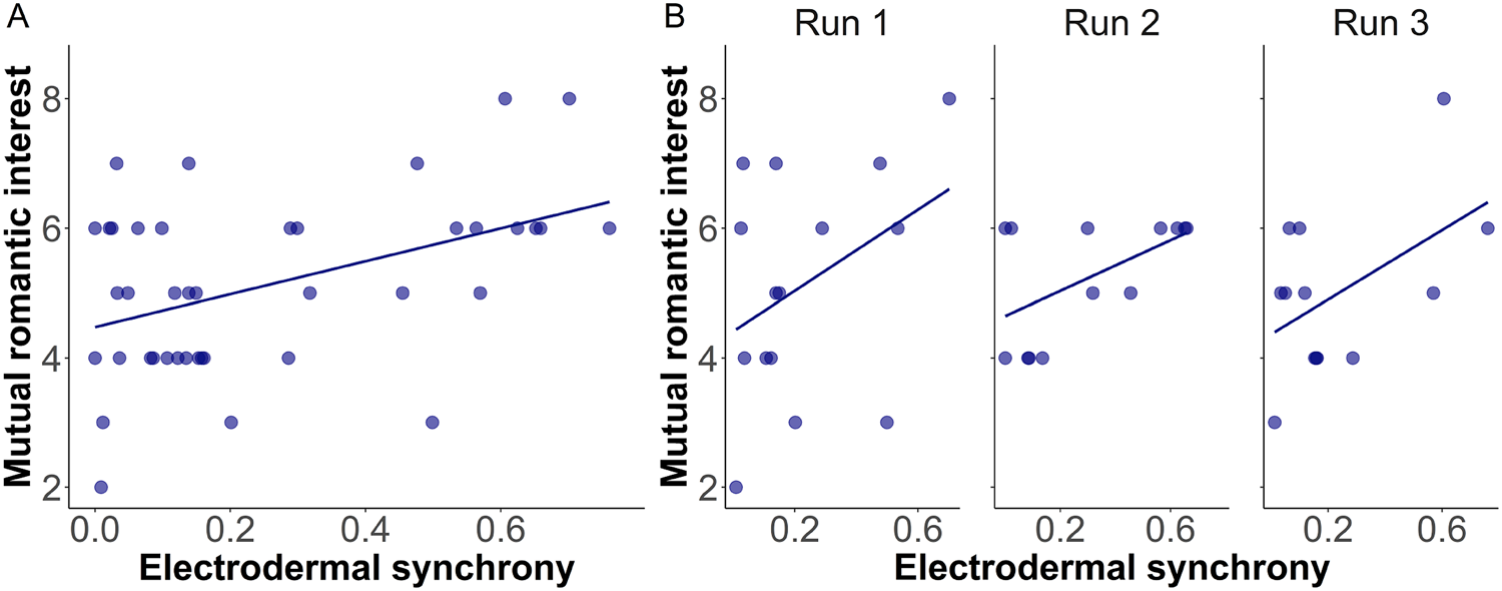
Increased electrodermal synchrony during a date is associated with increased romantic interest. (**A**) Dyadic electrodermal synchrony between partners in a date (x-axis), is associated with increased mutual romantic interest (y-axis), multilevel model analysis: β = 2.83, *p* = 0.006; Pearson r = 0.51, 95% confidence interval = [0.23, 0.72], *p* < 0.001, BF_10_ = 73.23, N = 42 dates, combined across three experimental runs (run 1, N = 15 dates, run 2 N = 14 dates, run 3 N = 13 dates). (**B**) The results in each experimental run.; Mutual romantic interest is calculated as the sum of the man’s and the woman’s ratings of romantic interest in each other (see “Methods”).

Synchrony in electrodermal activity between a man and a woman during a date significantly correlated with mutual romantic interest (multilevel model analysis: β = 2.83, *p* = 0.006; Pearson r = 0.51, 95% confidence interval = [0.23, 0.72], *p* < 0.001, BF_10_ = 73.23). This effect is calculated on data combined from three runs^44^ because it was replicated across three runs with different participants (Fig. 2).

### A bio-behavioral marker for date-outcome

**Figure 3:**
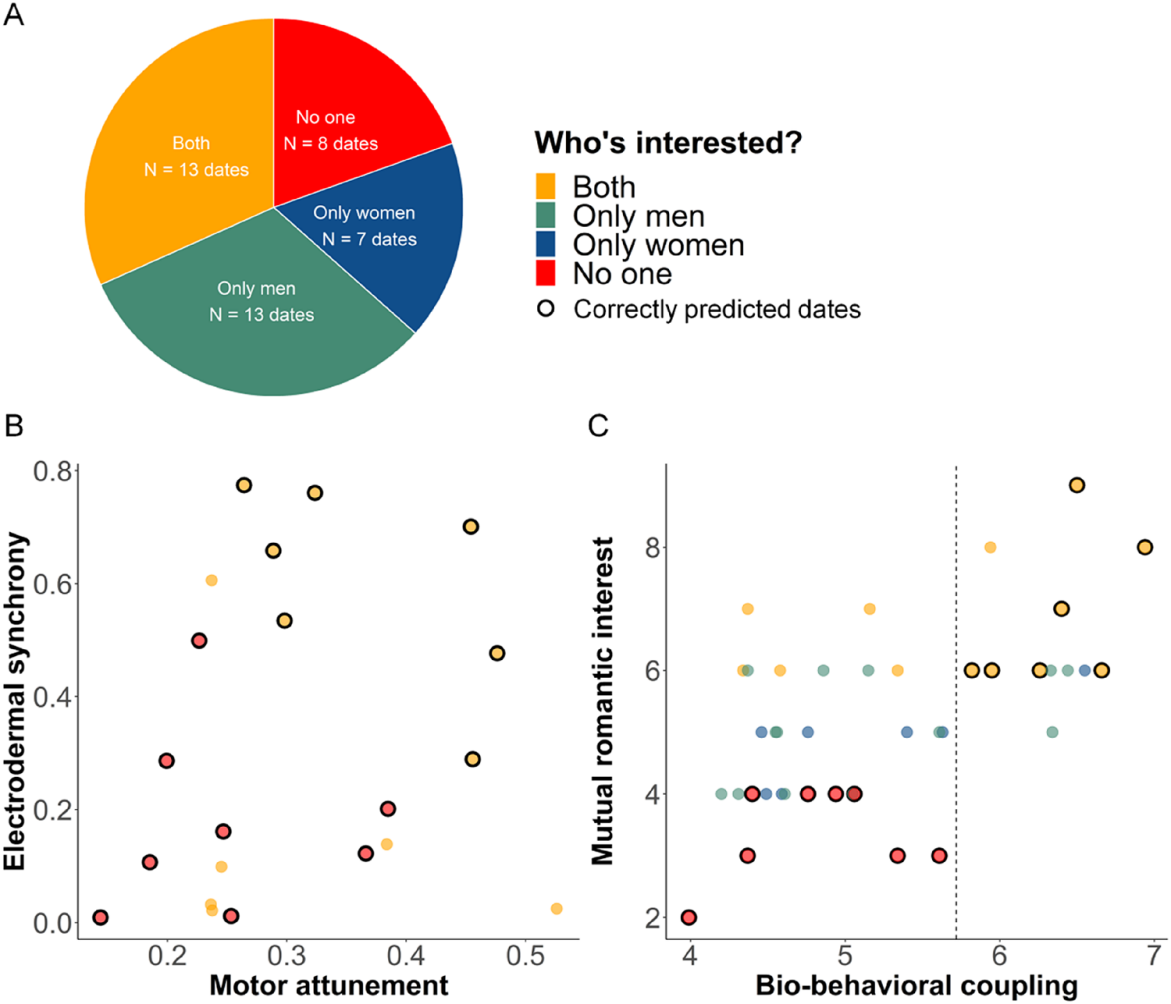
Coupling of electrodermal activity and behavior during a date indicates the date-outcome. (**A**) The date outcome was calculated based on the romantic interest of both partners. After each date, subjects rated the romantic interest in their partner on a scale of 1 to 5. Based on the median of the ratings, participants who rated their partner with a score of 3 or above were considered interested. (**B**) The dates’ outcome as a function of motor attunement (x-axis) and electrodermal synchrony (y-axis). Electrodermal synchrony and motor attunement are positively associated (Pearson’s r = 0.304, 95% confidence interval = [0.05, 0.57], *p* < 0.027). Moreover, a multilevel model analysis shows that both electrodermal synchrony (β = 2.75, *p* < 0.001) and motor attunement (β = 4.39, *p* = 0.003) are significant predictors of mutual romantic interest. With a leave-one-out linear regression classifier, the model correctly classified 71% of the successful dates (‘both interested’, in yellow) and unsuccessful dates (‘no one interested’, in red). Correctly predicted dates are marked with a black circle. (**C**) A linear regression showing the association between bio-behavioral coupling (i.e., electrodermal synchrony and motor attunement, x-axis) to ratings of mutual romantic interest (y-axis) (Pearson’s r = 0.56; *p* < 0.001, BF_10_ = 220.96). The dashed line depicts the threshold between successful and unsuccessful dates that enables optimal prediction of the date outcome, calculated with Receiver Operating Characteristic (ROC) curve (see “Methods”).

In addition to electrodermal synchrony, we also assessed the motor attunement during the interaction using pixel-by-pixel video analysis and computed cross-correlations in body movements (see “Methods”). A multilevel model analysis shows that both electrodermal synchrony (β = 2.75, *p* < 0.001) and motor attunement (β = 4.39, *p* = 0.003) are significantly associated with mutual romantic interest. We then computed a *bio-behavioral* measure, combining electrodermal synchrony with motor attunement (henceforth marked as *bio-behavioral coupling)* using linear regression, to test the extent to which *bio-behavioral coupling* predicts the date-outcome (Fig. 3). Next, we applied a linear regression model with a leave-one-out procedure. We predicted the outcome of each date, by calculating a linear regression model that includes all other data points, and not the predicted date. The model used both electrodermal synchrony and motor attunement, in order to predict whether the partners were attracted to one another in each date. Our findings show that the combined measures of bio-behavioral coupling correctly predicted the outcome of 71% of the successful dates (‘both interested’) and unsuccessful dates (‘no one interested’) (Fig. 3), which is significantly different from chance (H_0_ ~ Binomial(21, 0.5), *p* = 0.013). This provides evidence for a role of electrodermal synchronization and behavioral attunement in romantic attraction. While synchrony is not a necessary condition for romantic interest—it is indicative of it: if bio-behavioral coupling during a date is high, the romantic interest of both dyadic partners during this date is high as well. The association between motor attunement by itself and mutual romantic interest showed a moderate correlation and was marginally significant (r = 0.233, *p* = 0.06).

### Bio-behavioral coupling explains the variance of romantic interest over and above physical appearance

We estimated the contribution of bio-behavioral coupling in explaining the variance in mutual romantic interest, over and above physical appearance using a multilevel model analysis. Bio-behavioral coupling significantly predicted the mutual romantic interest (β = 3.66, *p* < 0.007), when controlling for the random effects of both the run number and participant. Physical appearance of men marginally predicted the date outcome (β = 0.39, *p* < 0.069), and significantly in women (β = 0.52, *p* < 0.005), when controlling for the random effects of both the effects of run number and participant.

Bio-behavioral coupling is also a significant predictor of mutual interest when using a linear model. When controlling for the physical appearance of women and men, both electrodermal synchrony (β = 8.00, *p* = 0.032) and motor attunement (β = 10.30, *p* = 0.019) are significant predictors of mutual romantic interest (partial correlation, Pearson r = 0.61, 95% confidence interval = [0.41, 0.80], *p* < 0.001, BF_10_ = 37.29). Physical appearance on its own in both genders explains 24.7% of the variability (Pearson r = 0.50, *p* = 0.019). Computing a bio-behavioral marker of coupling significantly improves the linear regression model well beyond physical appearance to 56.4% (Pearson r = 0.75, *p* < 0.001, BF_10_ = 1243.36). The contribution of bio-behavioral coupling in variance accounted for (Δ*R*^*2*^) equals 26.7%, which is significantly different from zero (*F*_(2, 21)_ = 6.43, *p* = 0.007).

### Gender differences

Having established a positive association between bio-behavioral coupling and mutual romantic interest, we further examined potential gender differences, by testing the preferences of women and men separately.

#### Synchrony and sexual desire

**Figure 4:**
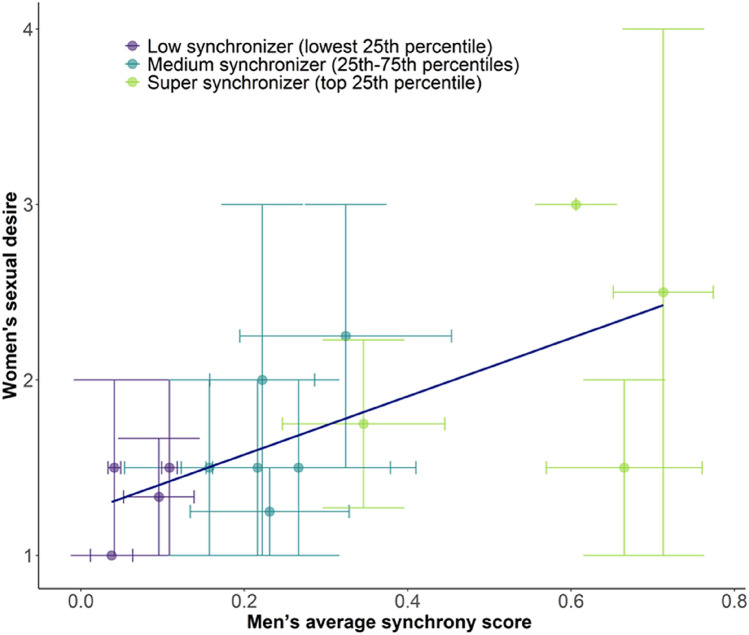
Women are more sexually attracted to synchronous men. We computed the correlation between participants' individual synchrony scores and their level of sexual attractiveness. The x-axis depicts mean electrodermal synchrony scores across men’s dates, while the y-axis depicts women’s sexual desire. Higher synchrony scores in men are associated with increased women’s sexual desire (Spearman r = 0.68, 95% confidence interval = [0.24, 0.93], *p* < 0.004, Bonferroni alpha = 0.025, BF_10_ = 7.55). Horizontal error bars depict the standard error of men’s mean synchrony, while vertical error bars depict the standard error of women’s mean sexual desire (see “Methods” section). For visualization: colors represent low synchronizers in purple (the lowest 25% synchrony scores of men), medium synchronizers in indigo (25–75% synchrony scores of men), and super synchronizers in green (top 25% synchrony scores of men).

Women’s mate-preference was largely associated with electrodermal synchrony. Specifically, electrodermal synchrony during the date significantly correlated with women’s sexual desire (multilevel model analysis: β = 2.27, *p* = 0.019; Spearman ρ = 0.38, 95% confidence interval = [0.06, 0.61], *p* = 0.012, Bonferroni alpha = 0.025, BF_10_ = 31.42). This effect was not evident in men (multilevel model analysis: β = 0.07, *p* = 0.933; Spearman ρ = − 0.15, 95% confidence interval = [− 0.45, 0.19], *p* = 0.335, BF_01_ = 2.20). Moreover, since every subject participated in multiple dates, we computed the average electrodermal synchrony scores of each participant across all of their dates. Then, we tested if individual synchrony scores are associated with sexual attractiveness. A multilevel model analysis revealed a significant gender and synchrony interaction, which indicates that women are more attracted to synchronous men, than men are attracted to synchronous women (β = 3.12, *p* = 0.037). Similarly, Spearman correlation show that women are sexually more attracted to men who are better synchronizers (Spearman ρ = 0.68, 95% confidence interval = [0.24, 0.93], *p* = 0.004, Bonferroni alpha = 0.025, BF_10_ = 7.55, Fig. 4). This effect was not evident in men (Spearman ρ = − 0.29, 95% confidence interval = [− 0.71, 0.16], *p* < 0.300, BF_10_ = 1.127).

## Discussion

Our findings demonstrate that bio-behavioral coupling during a date predicts romantic interest and sexual attraction in humans. This study shows that successful dates, which resulted in mutual romantic interest, are characterized by increased electrodermal synchrony and attunement of behavior. When a man and a woman are highly synchronous and attuned during a date, their mutual romantic and sexual interest are high as well. This provides evidence that sexual and romantic attraction in humans involve social adjustment of the sympathetic nervous system and motor behaviors. Moreover, the results point that individuals who are better at adapting their physiology and behavior to their partner during the date, are more likely to attract a partner. This suggests that adaptation of physiology and behavior to a partner during interaction can promote romantic bonding.

Synchronization of activity in the sympathetic nervous system during human interaction could reflect a social effect on the regulation of arousal and affect (i.e., ‘social regulation’). The ability to tap onto another human’s physiology is a central evolutionary feature that supports survival in social animals^25^. Social regulation is vital in early-life, as infants rely on their caregivers to regulate most aspects of their metabolic processes, including energy expenditure, immunity, temperature and arousal^25–27^. Despite gradual weaning from the complete dependence on social regulation, adults’ physiology and behavior are continually predisposed to be regulated by others, in particular—close others^37,46–48^. Multiple physiological processes are socially regulated through synchronization among romantic partners, from autonomic processes such as arousal^35^, respiration and blood pressure^49–51^, through endocrine processes marked by plasma levels of hormones as cortisol and oxytocin^52,53^, to neural processing measured with electroencephalogram (EEG)^29,54^ and Functional Near-Infrared Spectroscopy (fNIRS)^55^. Through bio-behavioral social regulation, metabolism is optimized during interactions^25^. Thus, bio-behavioral coupling during a date may be indicative for the efficacy of social regulation, marking an adaptive fit between a male and a female.

While synchrony is a dyadic phenomenon, constructed in time by both partners, it is not equally associated with sexual preferences among men and women. This study reveals gender differences in sexual attraction: women desire high-synchrony men, more so than men desire high-synchrony women. Several studies report a gender-difference in the involvement of the sympathetic nervous system in sexual arousal, which is stronger in females^56,57^. This is in line with the stronger association found between women’s sexual desire and synchrony in electrodermal activity, a sympathetic measurement. This gender difference suggests that classic evolutionary theories, which couple sexual desire with reproduction, seem more relevant to males, and underestimate the role of social interaction and cooperation in sexual selection^19,20^. In women and other female primates sexual desire is uncoupled from the reproduction, and sexual interactions occur throughout the menstrual cycle^58^. As such, the purpose of sexual and romantic desire in primates cannot be limited to fertility, and can serve an additional social purpose of bonding^59^. Whereas physical appearance is a well-studied feature in mate selection^15,16^, this study points to an additional mechanism, co-regulation of physiology and behavior, which impacts mate selection beyond physical appearance in both males and females.

When assessing dyadic interaction, not only synchrony (i.e., the instantaneous matching between partners) is relevant for affiliation, but also the sequential impact partners have on each other, or attunement^37,60,61^. Being sensitized to the partner’s behavioral cues, and attuned adjustment of behavior in response to those cues, are key features in social interaction, bonding and closeness^36,40,62^. Synchrony and attunement are complementary features in dyadic interaction, together enabling both simultaneous resonance and attuned responsiveness, and can maximize the social impact on the regulation of physiology during interaction. Indeed, it is the combination of both synchrony and attunement (measured here as ‘bio-behavioral coupling’) that best predicted the partners’ attraction. This research found a positive association between electrodermal synchrony and motor attunement. Future research is needed to test whether interactive flexibility serves as a behavioral mechanism to achieve physiological synchrony.

Previous research on speed dating characterized verbal and nonverbal parameters that are associated with attraction and romantic interest^63–67^. Yet, this is the first study to our knowledge that combines dynamic measures of naturally occurring behavior and physiology during a first date, to assess the theoretical idea that bio-behavioral adaptation to a romantic partner can serve as means for co-regulation and promote romantic and sexual attraction. The theoretical idea that bio-behavioral synchrony is a strategy for co-regulation, which promotes attachment has been extensively studied in the parent-infant bond^21,68^. Here we show evidence supporting a similar mechanism in romantic bonds, whereby high bio-behavioral coupling during a date is indicative that both partners are interested in each other.

It is important to note that we cannot determine the causality direction between bio-behavioral coupling and romantic or sexual preferences. It is possible that bio-behavioral synchrony increases attraction, or that feeling attracted improves the ad-hoc motivation to synchronize. Another unknown is whether the extent of synchrony during a date reflects an a-priori compatibility between the partners, or an individual “synchrony-aptitude” that enables to adapt to the partner in order to attract them. The study design, in which each participant met with multiple partners, provided evidence that some individuals tend to synchronize with their partner more than others, regardless of the partner and across multiple partners. Moreover, these synchronous individuals are found to be more attractive. This indicates that rather than a-priori compatibility, sexual and romantic attraction is potentially determined by the ability to adapt to the partner. Nonetheless, future mechanistic studies in adults are needed to assess the causal effects of synchrony and partner selection. Future studies are warranted to test these hypotheses in same-sex couples, and other cultures as well.

In particular, given the gender differences, sexual and romantic attraction in women-women bonds and men-men bonds can shed light on the role of sexual and romantic desire as a potential mechanism of bonding, beyond the purpose of fertility. To conclude, our findings provide evidence associating biological synchrony and behavioral attunement with romantic and sexual attraction. This suggests that co-regulation of bio-behavioral rhythms during interaction might function as a mechanism for mate-selection in humans.

## Methods

### Participants

Thirty-two undergraduate students (16 women), with an age range of 21–28 (Mean = 24, SD = 1.7 years), participated in the experiment. Participants were recruited via the university online experiment system. All participants were native Hebrew speakers, heterosexual, cis-gender , single, and interested in a romantic relationship. Participants were remunerated for their participation. The experiment was approved by the ethical committee of the Faculty of Social Sciences of the Hebrew University of Jerusalem and performed in accordance with relevant guidelines and regulations. Each participant signed an informed consent form prior to participation.

### Procedure

The experiment was conducted in three experimental runs of sixteen dates, in which a male and a female met for a five-minute date. In the first and second runs, 4 female-participants and 4 male-participants were invited to the lab, resulting in 16 dates. The third run consisted of four rounds (2 female-participants and 2 male-participants in each round). Overall, the cohort includes 48 dates, and two dates were excluded from analysis because the partners were formerly acquainted. Dates occurred one at a time in a dedicated room. During the dates, we recorded the subjects’ electrodermal activity at frequency of 4 Hz using an Empatica E4 wristband. Moreover, we video-recorded the dates and conducted an automated video-analysis to infer the motion of each partner during the date pixel-by-pixel, frame-by-frame at a frequency of 10 Hz.

Acquisition of behavioral data from one date and physiological data from four dates was not completed due to equipment malfunction and were not included in the analyses. Thus, while all behavioral analyses were conducted on forty-five dates, all physiological analyses were based on forty-two dates, and physical appearance ratings were not provided by women in ten dates and men in six dates.

Before and in between dates, each participant was assigned a private waiting room. While waiting, participants did not have access to their smartphones. Participants refrained from caffeinated and/or sugared food and beverages. Upon date onset, the experimenter escorted subjects to the date room. Immediately after each date, subjects returned to their private waiting room where they rated their romantic interest and sexual attraction to the partner, and their physical appearance.

### Sample size and power

When there are multiple sources of random variability in a design, the most accurate method of determining the power is simulations that capitalize on random effects revealed in actual data (see Finkel et al., 2015)^69^. Thus, to estimate the sample size, we ran an initial sample (run 1), and used a bootstrapped power calculation. In this run, each subject participated in 4 dates, giving a total of 16 dates. Using bootstrap sampling (n = 10,000), we found that adding two more rounds to our experiment (i.e., n = 48), would yield a statistical power of ~ 77%. Hence, we stopped data collection after three runs and excluded dates with no physiological data, leaving 42 valid dates. Additionally, according to previous studies on relationship science (Finkel et al., 2015)^69^, sensitivity analysis indicates that the detectable effect size with the current sample size is ~ 0.3 and above at alpha of 0.05 and power of 0.8^63,70^. Given the additional sources of variance due to the multilevel design (participant, date, run), we also estimated the statistical power of the entire sample across three runs by calculating the post-hoc power for the multilevel model using the powerSim function (from the simr package) in R 3.6.1^71^. This analysis revealed a power of 73% to find a significant effect of electrodermal synchrony on romantic interest (as reported in Fig. 2).

### Data acquisition and analyses

#### Behavior

We applied automated video analysis to infer the motion of each partner during the date^37^. From each video, we separated the images to two regions, each one containing one partner of the dyadic interaction. We then extracted the velocity of each pixel in each frame (using the optical flow algorithm^72^), and summed their squared values over all pixels to assess the total motion at each moment of each partner during the date. Thus, for each participant we have a measure for the total motion he/she did during each time point in the date (sampled at 10 Hz).

#### Physiology

During the five-minute date, we measured participants’ electrodermal activity using Empatica E4 wristbands. Electrodermal activity refers to the continuous variation in the electrical characteristics of the skin. Typically, it is measured as skin conductance by applying a small, constant voltage to the skin. As the *voltage* is kept constant, *skin conductance* can be calculated by measuring the current flow through the electrodes. Electrodermal activity is controlled by the sympathetic nervous system and reflects the level of arousal and orientation of attention^42,43^. Electrodermal activity has been reported to be sensitive to social stimuli and reactive during social interactions^73,74^, and specifically between romantic partners^35^. Thus, electrodermal activity is a key target to assess autonomic synchrony in a romantic context.

The E4 wristband is a wearable and wireless device. We placed the wristbands on the inner wrist of participants’ right hand^75^, and recorded the electrodermal signal throughout the date. Then, to calculate electrodermal synchrony, the electrodermal signals from both partners were temporally aligned using a global timestamp, marking the beginning of each date^76,77^.

The wristbands contain an electrodermal activity sensor with sampling frequency of 4 Hz, resolution of one digit –900 pSiemens, range of 0.01–100 μSiemens, and alternating current (8 Hz frequency) with a maximum peak to peak value of 100 μAmps (at 100 μSiemens). The obtained electrodermal signal was streamed via Bluetooth to an E4 App for iOS/Android for online control. Subjects were wearing the E4 devices for at least one minute prior to starting the date, to ensure proper sensor calibration^76^.

Previous studies reported the use of Empatica E4 wristbands in behavioral experiments that measured electrodermal activity^78–80^. Important advantages of the E4 wristbands is that they are quick to connect and wireless. Hence, unlike electrode-based-devices that measure electrodermal activity, they do not interfere with natural behavior in experimental settings, enhancing the ecological validity of the obtained results. However, since this method is not the gold-standard for measuring electrodermal activity, we conducted a separate experiment to validate the E4 signal. Specifically, we compared the output from the Empatica E4 wristbands to the skin conductance output of an Atlas constant voltage system (0.5 V ASR Atlas Researches, Hod Hasharon, Israel). The Atlas system has been used in dozens of physiological experiments over the last 2 decades^81–83^. Importantly, our validation experiment is consistent with previous research validating the E4 device^84–87^, providing support for the validity of the Empatica wristbands (see Supplementary Figure S1, as well as Supplementary Results Section for a full description of the Empatica E4 validation experiment).

See the Supplementary Results for detailed description of each date's electrodermal activity (Figure S5) and motion data (Figure S6), as well as descriptive statistics (Table S1).

#### Computing synchrony

Synchrony was computed using Matlab and R 3.6.1^88^. We computed two dyadic measures that reflect the bio-behavioral interactive transactions between men and women during the date: Dyadic Synchrony and Dyadic Attunement.

**Figure 5:**
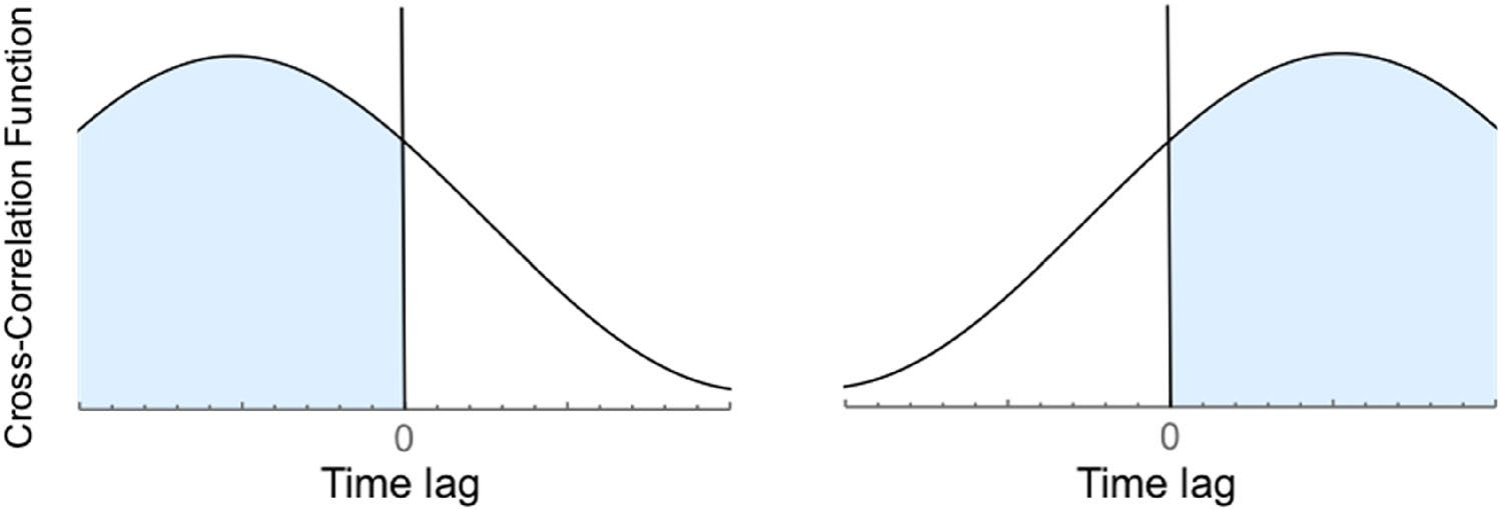
Cross-correlation values at different time-lags indicate dominance in leadership. In this graphical scheme, when summing across all time-lags in a cross-correlation analysis in a specific window, either the woman leads the dyadic interaction, or the man. When most of the mass of the cross-correlation function is on the negative side (left plot), the woman is the dominant leader in this time window. When most of the mass is on the positive side (right plot), the man is the dominant leader in the time window. We computed the dominance in leadership per each sliding window to assess the turn-taking in interactive leadership, for participants’ motion and physiology.

1. Calculating dyadic synchrony—Synchrony reflects partners alignment of behavior or physiology at the same time (i.e., the cross-correlation at lag 0). For each date, we calculated dyadic synchrony in electrodermal activity and behavioral motion. Correlations were squared in order to account for interpersonal coordination (i.e., in-phase and anti-phase synchrony)^89^.
2. Calculating dyadic attunement—in addition to synchronization, we also assessed the motor attunement of partners to each other by testing who leads the interaction and who follows. To trace changes in leader–follower turn-taking, we divided each time-series into ten second windows with five seconds offset. Then, we computed the cross-correlation of motion in each window. The cross-correlation function indicates the level of correlation at different time-lags. Synchrony at positive time-lags indicates leadership of one partner, while synchrony at negative time-lags indicated leadership of the other partner. We then summed across all time-lags to indicate the dominance in leadership in that specific time-window (i.e., the center of mass of the cross-correlation function at that specific time-window; the cross-correlation function is given by: $\mathrm{c}\left(\uptau \right)=\sum_{\mathrm{n}=0}^{\mathrm{N}-\uptau -1}{\mathrm{E}}_{\mathrm{woman}}\left(\mathrm{n}+\uptau \right){\mathrm{E}}_{\mathrm{man}}\left(\mathrm{n}\right)/\mathrm{ std}\left({\mathrm{E}}_{\mathrm{woman}}\right)\mathrm{std}\left({\mathrm{E}}_{\mathrm{man}}\right)$, where ${\mathrm{E}}_{\mathrm{woman}}(\mathrm{n})$ is the woman’s motion and ${\mathrm{E}}_{\mathrm{man}}(\mathrm{n})$ is the man’s motion at frame\sample n (see Fig. 5). The variance of this parameter, across all time-windows, indicates leader–follower turn-taking, meaning, the flexibility of motion leadership across the date and thus indicates the attunement of the partners in the date. Attunement was similarly calculated for the electrodermal activity data.

### Romantic ratings

After each date, each participant rated the date on three measures on a scale of one to five: romantic interest in the partner, sexual attraction to the partner, and the physical appearance of the partner. Thus, for each participant per each date, we calculated the personal scores of *Romantic Interest in partner, Sexual Attraction to partner,* and *Physical Appearance of partner*. Moreover, a dyadic variable of *Mutual Romantic Interest* was computed as the sum of Romantic Interest of both men and women. Romantic interest did not differ across the three experimental runs (see Supplementary Figure S2). We separated the dates to those where both or none of the men and women were interested (romantic ratings correlation between partners: rho = 0.69; *p* < 0.001), and where only men or only the women were interested (romantic ratings correlation between partners: rho = − 0.05; *p* = 0.372). It was recently demonstrated that the extent of dyadic reciprocity in the romantic interest between the man and the woman in the date is essential for predicting dyadic bio-behavioral measures^90^.

### Testing the associations between synchrony and date ratings

#### The association between synchrony and romantic interest

To model the synchrony across the dates, we first computed the time course of synchrony using a sliding window. We found that successful dates are characterized by increased electrodermal synchrony in the first two minutes (see Supplementary Figure S3). Therefore, we computed electrodermal synchrony across the first two minutes and applied Pearson correlations to test the association between the dyadic measures and romantic interest. We computed bootstrapped *confidence intervals* with *1000 iterations.* While the most significant differences were found in the first two minutes, calculating electrodermal synchrony across the entire 5-min is also significantly associated with romantic interest (see supplementary Figure S4). Analyses were corrected for multiple hypotheses testing based on four independent variables: Motion Synchrony; Motion Attunement; Electrodermal Synchrony; Electrodermal attunement.

#### The predictive power of bio-behavioral coupling on date success

We applied linear regression, and a leave-one-out procedure^91^, to assess the predictive power of the bio-behavioral synchrony during the date on the romantic ratings after the date. Specifically, for each date, we calculated a linear regression model which includes all data points except that date. The regression model then uses electrodermal synchrony and motor attunement to predict the romantic interest score of the remaining date. This procedure was repeated twenty-one times (which equals the total number of successful and unsuccessful dates).

The prediction scores were used to construct a Receiver Operating Characteristic (ROC) curve. This curve is commonly used in binary classification decisions. It describes the performance of our regression model across all discrimination thresholds, by plotting the true positive rates (i.e., proportion of dates where both partners were interested is classified as ‘both interested’) and false positive rates (i.e., proportion of dates where both partners were not interested is classified ‘both interested’). We then selected the optimal threshold—i.e., the point on the curve which provides the best separation between successful dates (‘both interested’) and unsuccessful dates (‘no one interested’) and predicted the outcome of each date.

#### The association between synchrony and sexual desire

##### Calculating a personal synchrony score

Since every subject participated in multiple dates, we computed the average dyadic coupling scores for each participant. We used Spearman correlation to test if high synchrony scores are associated with sexual attraction.

#### Multilevel model analyses

Whenever mentioned in the main text, dyadic data were analyzed using multilevel models^44,70,92^. These models account for the statistical non-independence of the data points as individuals participated in several dates and belonged to three different runs. The models controlled for the random effects of recurring data from individuals that repeated in different dates and for their nesting in specific runs. For bio-behavioral coupling, we used a model with fixed effects for bio-behavioral coupling and physical appearance, while accounting for the random effects for the intercept of recurring data from individuals that repeated in different dates and for their nesting in specific runs. See code for each calculation at https://osf.io/5b246/.

#### Bayesian analyses

To augment the classical statistical inference (i.e., correlations, linear regression, and multilevel model analyses), we also included Bayesian analyses and computed Jeffreys-Zellener-Siow (JZS) Bayes Factors (BFs). The default prior settings (used by R) were left unchanged. BF values ≥ 3 is considered to provide substantial to moderate support for the alternative hypothesis^93^. For all correlations, either the BF_10_ (favoring the alternative hypothesis) or the BF_01_ (favoring the null hypothesis) is reported.

All plots were created using the ggplot2 package50 in R 3.6.1^45^. 

## Supplementary Information

Supplementary Information.

## Data Availability

All data, as well as the analyses scripts, were uploaded to a public repository at https://osf.io/5b246/ and can become available upon request.
