# Supplementary material for: Bio-behavioral synchrony is a potential mechanism for mate selection in humans
Source: Sci Rep. 2022 Mar 21;12:4786. doi: 10.1038/s41598-022-08582-6 (PMC8938461; doi:10.1038/s41598-022-08582-6)
Supplement: Supplementary file 1 — Supplementary Information. [file 41598_2022_8582_MOESM1_ESM.docx]

Bio-Behavioral Synchrony is a Potential Mechanism for Mate Selection in Humans

Lior Zeevi^1^†, Nathalie klein Selle^1^†, Eva Ludmilla Kellman^2^, Gal Boiman^1^, Yuval Hart^1^ and Shir Atzil^1^*

**Supplementary Results**

**Empatica wristbands are valid for the measurement of EDA**

A separate experiment (*N* = 10 dates) was conducted to validate the electrodermal activity (EDA) signal of our Empatica E4 wristbands. This was done by comparing the skin conductance output from the E4 wristbands to that of an Atlas constant voltage system (0.5V ASR Atlas Researches, Hod Hasharon, Israel). After attaching participants to both devices, they were explained about an upcoming concealed information test (CIT). Before starting the CIT, participants were presented with 6 cards and requested to choose 1. During the CIT, participants were presented again with all cards, in a serial manner, and requested to conceal their knowledge of the chosen card. Previous research has shown that the chosen (concealed) cards induce enhanced skin conductance responses compared to the non-chosen cards – also called the CIT effect (Klein Selle, Verschuere, Kindt, Meijer, & Ben‐Shakhar, 2016; Meijer & Verschuere, 2017). When correlating the CIT effects obtained from the Empatica and Atlas devices, a strong and positive association was found, Pearson’s r = 0.640 [0.02, 0.94], p *=* 0.023 (Figure S1). This result supports the validity of the Empatica wristbands.

**
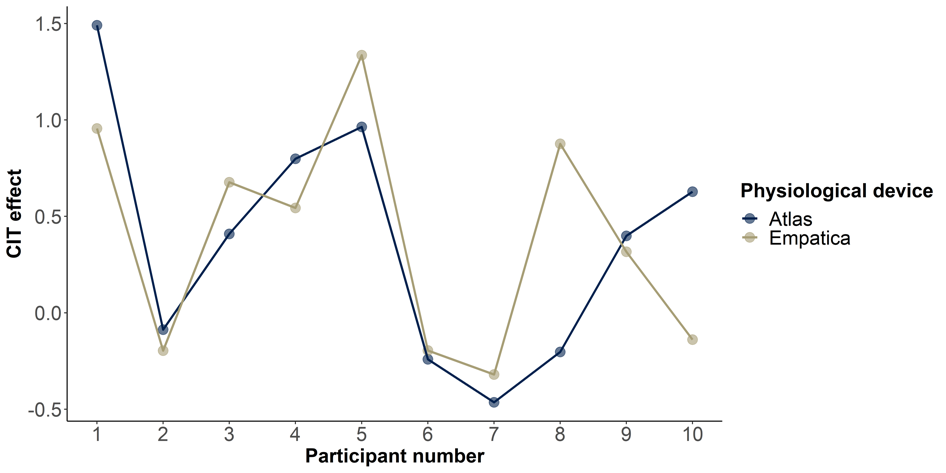
**

**Figure S1. Empatica wristbands are valid for the measurement of EDA.** When comparing CIT effects obtained using the Empatica and Atlas devices, a strong and positive association was found, Pearson’s r = 0.640 [0.02, 0.94], p = 0.023. This result supports the validity of the Empatica wristbands. The x-axis depicts participants number, while the y-axis depicts the CIT effect.

**Mutual romantic interest does not differ across experimental runs**

To confirm that there were no differences in romantic interest ratings across experimental runs, we ran an ANOVA analysis, demonstrating no significant difference across runs, which means that the ‘run number’ did not affect the date outcome (F = 1.01, p < 0.37) (Figure S2).

**
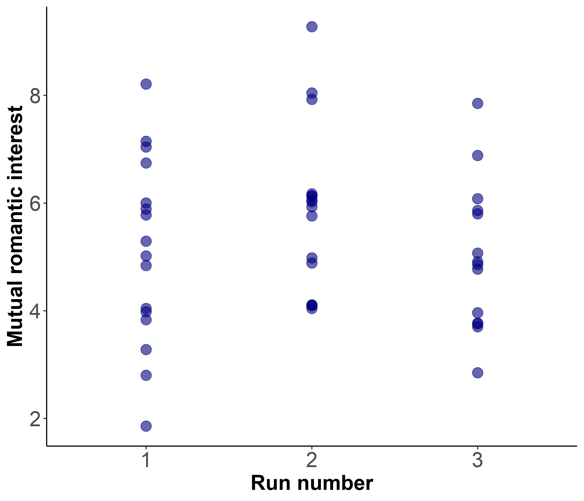
**

**Figure S2. Mutual romantic interest does not differ across experimental runs.** Mutual romantic interest scores, which were obtained after each date, were similar across three experimental runs. The x-axis depicts run number, while the y-axis depicts mutual romantic interest scores. Vertical jitter was added for clarity of presentation.

**Electrodermal synchrony is highest during the first 2 minutes of successful dates**

Electrodermal Synchrony is highest during the first two minutes of successful dates (i.e., both partners are romantically interested), possibly due to habituation of the skin conductance signal over time (Figure S3). Hence, for our main analyses (as reported in the paper), differences in electrodermal synchrony are calculated during the first two minutes of a date.

**
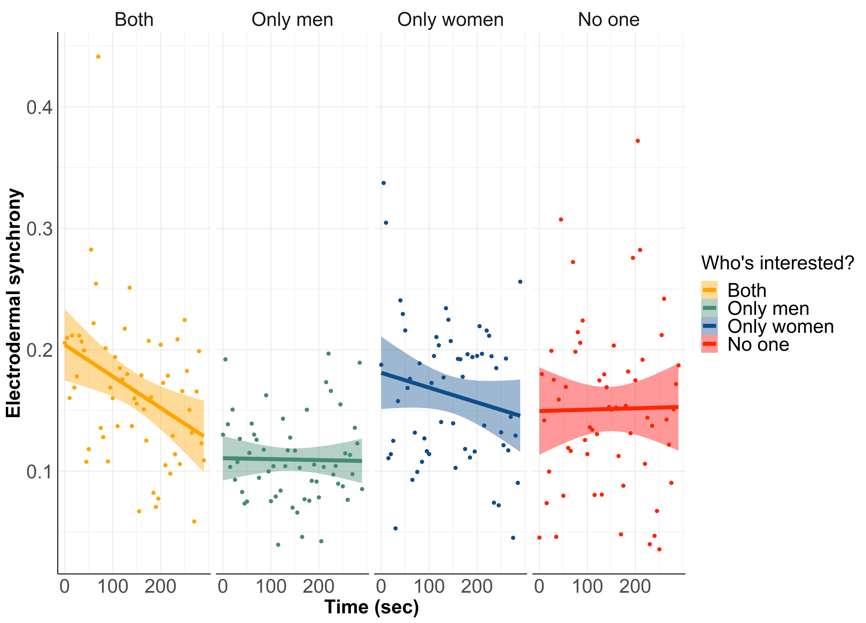
**

**Figure S3.** **Successful dates are characterized by increased electrodermal synchrony in the first two minutes.** When both partners are romantically interested in each other (yellow scatter plot), electrodermal synchrony is highest at the beginning of the date.

**Electrodermal synchrony during the entire 5-minutes date is associated with romantic interest**

Despite the fact that synchrony during the initial two minutes of the date was mostly predictive of romantic interest (as seen in Figure 2), Electrodermal Synchrony during the entire date also correlates significantly with romantic interest, Pearson’s r = 0.410 [0.17, 0.62], p < 0.007 (see Figure S4).

**
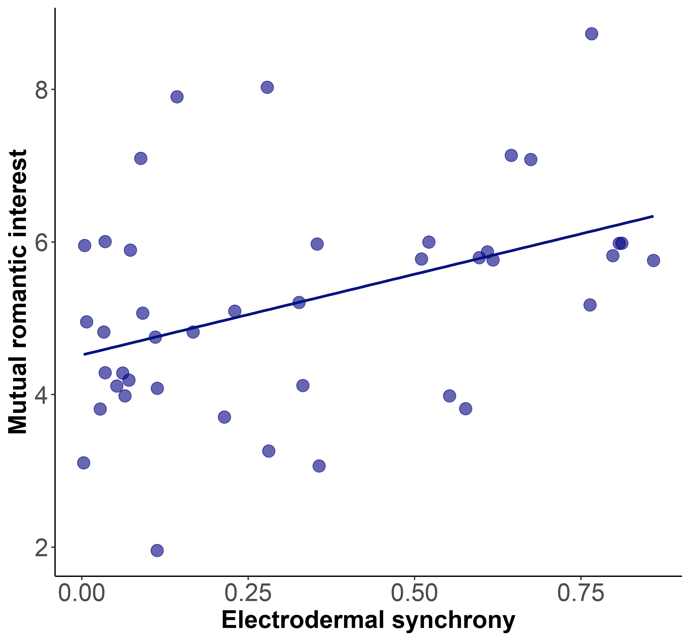
**

**Figure S4. Increased electrodermal synchrony during the entire 5-minutes date is associated with increased romantic interest**. Electrodermal synchrony in electrodermal activity (EDA), between partners in a date, is associated with increased mutual romantic interest. Pearson’s r = 0.410 [0.17, 0.62], p < 0.007. Mutual romantic interest is calculated as the sum of the man’s and the woman’s ratings of romantic interest in each other (see Methods)

**Patterns of individual electrodermal activity during the dates**

**Figure S5: Raw EDA data according to the "who's interested" categories:** Electrodermal Activity (EDA) is presented for men (green) and women (purple) during individual dates. The x-axis represents the progress, in seconds, of the five-minute dates. The y-axis represents EDA, measured by microSeimens units in the sampling rate of 4Hz, providing 1200 data points. Please see Table S1 for descriptive statistics for each participant.

**Patterns of individual motion behavior during the dates**

**Figure S6: Raw motion data according to the who's interested categories:** Motion data is presented for men (brown) and women (yellow) during individual dates. The x-axis represents the progress, in seconds, of the five-minute dates. The y-axis represents motion energy, measured by extracting the velocity of each pixel in each frame and summing their squared values over all pixels at a sampling rate of 10Hz, resulting in 3000 data points. Please see Table S1 for descriptive statistics for each participant.

**Table S1: Statistics of EDA and motion for each participant**

|  |  | **Personal Average EDA** | **Personal Variance EDA** | **Date Correlation sqr EDA** | **Personal Average Motion** | **Personal Variance Motion** | **Personal Correlation EDA-motion** |
| --- | --- | --- | --- | --- | --- | --- | --- |
| **Date1** | Woman | 1.12661 | 0.03975 | 0.51158 | 88.53 | 67958.7 | 0.031 |
|  | Man | 3.34509 | 10.9205 |  | 242.71 | 786983 | -0.130 |
| **Date2** | Woman | 0.13196 | 0.00222 | 0.00304 | 22.74 | 128191 | 0.081 |
|  | Man | 7.0689 | 0.31451 |  | 233.76 | 538010 | -0.078 |
| **Date3** | Woman | 0.13645 | 0.00112 | 0.64497 | 203.93 | 188237 | -0.119 |
|  | Man | 1.8139 | 1.49894 |  | 307.1 | 749915 | -0.223 |
| **Date4** | Woman | 0.71007 | 0.00064 | 0.59685 | 168.64 | 439614 | 0.117 |
|  | Man | 0.4387 | 0.01971 |  | 110.11 | 75978.5 | -0.176 |
| **Date5** | Woman | 0.04548 | 0.00024 | 0.05163 | 58.45 | 70452.7 | -0.081 |
|  | Man | 0.69581 | 0.0103 |  | 316.35 | 313548 | -0.09 |
| **Date6** | Woman | 0.32222 | 0.06207 | 0.07292 | 7319.33 | 3.6E+08 | -0.018 |
|  | Man | 0.69006 | 0.01725 |  | 8484.39 | 1.6E+08 | 0.033 |
| **Date7** | Woman | 0.42151 | 0.00012 | 0.16623 | 97.94 | 63625.3 | -0.09 |
|  | Man | 0.51264 | 0.00665 |  | 395.92 | 1436965 | -0.015 |
| **Date8** | Woman | 0.13516 | 0.00231 | 0.27835 | 9838.74 | 4.3E+08 | -0.221 |
|  | Man | 0.48814 | 0.02723 |  | 7920.55 | 2.7E+08 | -0.418 |
| **Date9** | Woman | 0.08904 | 0.00069 | 0.67329 | 201.75 | 461109 | -0.033 |
|  | Man | 0.7214 | 0.0634 |  | 522.44 | 1242073 | -0.07 |
| **Date10** | Woman | 0.14349 | 0.00159 | 0.22993 | 300.206 | 567109 | -0.138 |
|  | Man | 1.91681 | 0.1406 |  | 236.02 | 388633 | 0.091 |
| **Date11** | Woman | 0.34784 | 0.0003 | 0.33141 | 147.35 | 111189 | -0.077 |
|  | Man | 0.61879 | 0.00033 |  | 253.16 | 413971 | -0.021 |
| **Date12** | Woman | 0.09736 | 0.00012 | 0.21408 | 129.22 | 105858 | -0.065 |
|  | Man | 0.38362 | 0.02179 |  | 466.78 | 667637 | -0.184 |
| **Date13** | Woman | NA | NA | NA | 10159.6 | 2.2E+08 | NA |
|  | Man | NA | NA | NA | 24685 | 1.5E+09 | NA |
| **Date14** | Woman | 0.33054 | 0.00016 | 0.35652 | 15133.3 | 3.1E+08 | -0.055 |
|  | Man | 0.32495 | 0.10927 |  | 13377.2 | 1.3E+08 | 0.301 |
| **Date15** | Woman | 0.12876 | 0.00462 | 0.11395 | 3958.21 | 5.9E+07 | -0.136 |
|  | Man | 0.70781 | 0.17298 |  | 23395.6 | 2.5E+09 | 0.007 |
| **Date16** | Woman | 0.0788 | 0.00014 | 0.08766 | 8926.4 | 1.5E+08 | -0.097 |
|  | Man | 0.43742 | 0.00018 |  | 27311 | 1.2E+09 | 0.049 |
| **Date17** | Woman | 2.99182 | 3.24663 | 0.85892 | 12100.8 | 5E+08 | -0.253 |
|  | Man | 0.45023 | 0.00904 |  | 822.5 | 7940601 | -0.178 |
| **Date18** | Woman | 2.70307 | 3.4654 | 0.61784 | 965.4 | 1.2E+07 | -0.27 |
|  | Man | 0.16588 | 0.0006 |  | 3310.98 | 9.1E+07 | -0.123 |
| **Date19** | Woman | 0.13324 | 0.00257 | 0.09148 | 2149.48 | 1.7E+07 | -0.069 |
|  | Man | 0.84512 | 0.03381 |  | 5075.9 | 4.6E+07 | 0.014 |
| **Date20** | Woman | 0.22102 | 0.00166 | 0.35427 | 1065.4 | 4330369 | -0.012 |
|  | Man | 0.29473 | 0.0107 |  | 6802.32 | 8.5E+07 | 0.056 |
| **Date21** | Woman | NA | NA | NA | 1347.55 | 2.2E+07 | NA |
|  | Man | NA | NA | NA | 2522.61 | 2.2E+07 | NA |
| **Date22** | Woman | 0.14283 | 0.00152 | 0.0281 | 7481.49 | 1E+08 | 0.08 |
|  | Man | 0.15113 | 1.3E-05 |  | NA | NA | -0.019 |
| **Date23** | Woman | 0.06983 | 0.00043 | 0.06064 | NA | NA | 0.05 |
|  | Man | 0.18188 | 0.00027 |  | 19377 | 4.2E+08 | 0.032 |
| **Date24** | Woman | 1.2553 | 0.24924 | 0.80734 | 6652.71 | 1.2E+08 | -0.202 |
|  | Man | 0.19341 | 0.00607 |  | 13187.8 | 2.6E+08 | 0.15 |
| **Date25** | Woman | NA | NA | NA | 6362.27 | 6.2E+07 | NA |
|  | Man | NA | NA | NA | 6423.19 | 8.4E+07 | NA |
| **Date26** | Woman | 1.09883 | 0.16867 | 0.11058 | 119.63 | 120499 | -0.218 |
|  | Man | 0.12296 | 3E-05 |  | 200.74 | 189069 | -0.069 |
| **Date 27** | Woman | 0.11595 | 0.00018 | 0.76579 | 116.81 | 80033.9 | -0.12 |
|  | Man | 0.31509 | 0.01654 |  | 161.54 | 172272 | 0.08 |
| **Date28** | Woman | 0.12876 | 0.00014 | 0.11369 | 81.48 | 61997.4 | -0.156 |
|  | Man | 0.11272 | 0.00012 |  | 673.26 | 1391283 | -0.074 |
| **Date29** | Woman | 2.84443 | 5.05508 | 0.79785 | 233.75 | 329642 | 0.008 |
|  | Man | 0.26514 | 0.00402 |  | 719.17 | 1512069 | -0.051 |
| **Date30** | Woman | 0.15694 | 4E-05 | 0.60893 | 177.98 | 330506 | 0.153 |
|  | Man | 0.12873 | 9.9E-05 |  | 586.74 | 3817387 | -0.014 |
| **Date31** | Woman | 0.45674 | 0.01202 | 0.07064 | 435.58 | 3621871 | 0.096 |
|  | Man | 0.27026 | 0.00336 |  | 1017.91 | 8480395 | 0.04 |
| **Date32** | Woman | 0.10442 | 2.1E-05 | 0.00434 | 253.35 | 197036 | -0.028 |
|  | Man | 0.15883 | 3E-05 |  | 770.96 | 1335422 | 0.005 |
| **Date33** | Woman | NA | NA | NA | 12174.6 | 2.2E+08 | NA |
|  | Man | NA | NA | NA | 17060.3 | 6E+08 | NA |
| **Dat2 34** | Woman | 3.34284 | 0.14896 | 0.03508 | 32681.9 | 8.6E+08 | -0.01 |
|  | Man | 0.2075 | 0.00579 |  | 24995.5 | 5.2E+08 | -0.203 |
| **Date35** | Woman | NA | NA | NA | NA | NA | NA |
|  | Man | NA | NA | NA | NA | NA | NA |
| **Date36** | Woman | 0.53159 | 0.03024 | 0.76394 | 10076.9 | 1.6E+08 | 0.08 |
|  | Man | 0.65038 | 0.10226 |  | 29550.6 | 1.1E+09 | -0.047 |
| **Date37** | Woman | 3.18177 | 0.00084 | 0.03286 | 22267.8 | 2.7E+08 | 0.074 |
|  | Man | 0.35867 | 0.00402 |  | 30538.8 | 9E+08 | -0.014 |
| **Date38** | Woman | 0.8273 | 0.12422 | 0.55293 | 23250.4 | 5.9E+08 | 0.127 |
|  | Man | 1.49334 | 0.17908 |  | 19900.6 | 5.5E+08 | 0.264 |
| **Date39** | Woman | 0.20947 | 0.00593 | 0.32625 | 68561.2 | 5.6E+09 | -0.056 |
|  | Man | 0.16204 | 0.00043 |  | 40234.4 | 1.8E+09 | -0.352 |
| **Date40** | Woman | 0.43448 | 0.31531 | 0.03506 | 25190.7 | 5E+08 | 0.271 |
|  | Man | 1.11803 | 0.08038 |  | 21388.8 | 4.8E+08 | 0.142 |
| **Date41** | Woman | NA | NA | NA | NA | NA | NA |
|  | Man | NA | NA | NA | NA | NA | NA |
| **Date42** | Woman | 4.294 | 0.22581 | 0.81135 | 17966.3 | 4.3E+08 | -0.053 |
|  | Man | 3.58403 | 8.241 |  | 31625.2 | 1.1E+09 | -0.001 |
| **Date43** | Woman | 4.98424 | 14.9367 | 0.14444 | 56018.2 | 2.9E+09 | -0.047 |
|  | Man | 0.92381 | 0.00012 |  | 24377.5 | 4.2E+08 | 0.031 |
| **Date44** | Woman | 3.91388 | 0.00096 | 0.00728 | 48085.4 | 2.6E+09 | -0.146 |
|  | Man | 0.55542 | 0.0009 |  | 37280.7 | 6.3E+08 | -0.058 |
| **Date45** | Woman | 0.12878 | 8.2E-07 | 0.0652 | 27353.6 | 6E+08 | -0.102 |
|  | Man | 0.25762 | 0.00632 |  | 12999.6 | 1.7E+08 | -0.07 |
| **Date46** | Woman | 0.19986 | 0.01847 | 0.52081 | 34474.8 | 1.3E+09 | 0.252 |
|  | Man | 0.41054 | 0.02513 |  | 30313.5 | 5.1E+08 | -0.042 |
| **Date47** | Woman | 0.87789 | 0.01702 | 0.57714 | 35599.7 | 1.1E+09 | 0.189 |
|  | Man | 0.40862 | 0.00402 |  | 13321.3 | 2E+08 | -0.393 |
| **Date48** | Woman | 1.43262 | 0.0625 | 0.28304 | 34803.1 | 1.7E+09 | -0.075 |
|  | Man | 0.14283 | 0.00213 |  | 33901.5 | 6.9E+08 | -0.06 |

klein Selle, N., Verschuere, B., Kindt, M., Meijer, E., & Ben‐Shakhar, G. (2016). Orienting versus inhibition in the Concealed Information Test: Different cognitive processes drive different physiological measures. *Psychophysiology, 53*(4), 579-590.

Meijer, E. H., & Verschuere, B. (2017). Deception detection based on neuroimaging: Better than the polygraph? *Journal of Forensic Radiology and Imaging, 8*, 17-21.
